# Programmed Death 1 Regulates Memory Phenotype CD4 T Cell Accumulation, Inhibits Expansion of the Effector Memory Phenotype Subset and Modulates Production of Effector Cytokines

**DOI:** 10.1371/journal.pone.0119200

**Published:** 2015-03-24

**Authors:** Joanna J. Charlton, Debbie Tsoukatou, Clio Mamalaki, Ioannis Chatzidakis

**Affiliations:** Institute of Molecular Biology and Biotechnology FORTH- Hellas, GR-70013 Heraklio, Crete, Greece; Jackson Laboratory, UNITED STATES

## Abstract

Memory phenotype CD4 T cells are found in normal mice and arise through response to environmental antigens or homeostatic mechanisms. The factors that regulate the homeostasis of memory phenotype CD4 cells are not clear. In the present study we demonstrate that there is a marked accumulation of memory phenotype CD4 cells, specifically of the effector memory (T_EM_) phenotype, in lymphoid organs and tissues of mice deficient for the negative co-stimulatory receptor programmed death 1 (PD-1). This can be correlated with decreased apoptosis but not with enhanced homeostatic turnover potential of these cells. PD-1 ablation increased the frequency of memory phenotype CD4 IFN-γ producers but decreased the respective frequency of IL-17A-producing cells. In particular, IFN-γ producers were more abundant but IL-17A producing cells were more scarce among PD-1 KO T_EM_-phenotype cells relative to WT. Transfer of peripheral naïve CD4 T cells suggested that accumulated PD-1 KO T_EM_-phenotype cells are of peripheral and not of thymic origin. This accumulation effect was mediated by CD4 cell-intrinsic mechanisms as shown by mixed bone marrow chimera experiments. Naïve PD-1 KO CD4 T cells gave rise to higher numbers of T_EM_-phenotype lymphopenia-induced proliferation memory cells. In conclusion, we provide evidence that PD-1 has an important role in determining the composition and functional aspects of memory phenotype CD4 T cell pool.

## Introduction

When naïve T cells encounter antigen in a specific manner, they react and mount an immune response which involves multiple rounds of proliferation and production of effector T cells. Only a small fraction of the responding cells survive to form memory T cells which are typically CD44^hi^ [1]. However, relatively large numbers of CD44^hi^ T cells are found in normal, unimmunized mice and have been termed “memory phenotype” (MP) T cells [1].

The mechanisms governing generation and maintenance of MP cells are mostly unclear mainly due to their heterogeneity. The fact that MP cells increase with age [1] supported the idea that it is the encounter with environmental antigens—innocuous and pathogenic- that drives their generation. However that cannot explain their existence in germ free mice [2]. Moreover, there seems to be a different etiology for CD8 and CD4 T cells, when considering MP differentiation. In particular, homeostatic proliferation has been suggested to drive differentiation of MP CD8 T cells in mice [3] whereas, cross- reactivity with environmental antigens is proposed to drive generation of virus-specific MP CD4 T cells in virus-unexposed humans [4]. CD44^hi^ T cells with MP cell properties can also arise after transfer of naïve T cells to lymphopenic recipients through a process termed lymphopenia-induced proliferation (LIP) during which the naive cells change in their phenotype and function to resemble memory cells [5].

True antigen-specific memory and MP CD8 and CD4 T cells are broadly divided to two subsets, central memory (T_CM_) and effector memory (T_EM_) [6]. Although memory T cell categorization has since been expanded, the T_CM_/T_EM_ dichotomy seems to be most useful in describing memory T cell properties [7]. T_EM_ cells are phenotypically characterized as CD44^hi^CD62L^lo^ and are preferentially situated in spleen, bone marrow and tissues, whereas T_CM_ cells are CD44^hi^CD62L^hi^ cells that preferably locate to lymph nodes [6]. However, in mice the T_CM_ subset accounts for only a small fraction of MP CD4 T cells [8,9]. CD4 T_EM_ cells have been recently involved in contributing to autoimmune diseases such as experimental autoimmune encephalomyelitis in mice, autoimmune diabetes, rheumatoid arthritis and systemic lupus erythematosus, although the antigen specificity of these cells is not clearly defined [10].

T cell co-stimulation is an important factor in determining MP CD4 T cell differentiation and balance between T_CM_ and T_EM_ subsets [11,12]. PD-1 is a negative co-stimulatory molecule of the CD28/CTLA-4 family which negatively regulates TCR signaling when engaged by one of its ligands, PD-ligand 1 and PD-ligand 2 [13,14]. PD-1 has a well established role in induction and maintenance of peripheral T cell tolerance as well as in host response against acute and chronic infections [13,15]. PD-1 is expressed in MP cells, especially on CD4 cells and predominantly in the T_EM_ subset [16,17]. We recently showed that PD-1 inhibits accumulation of functional CD8 T_EM_ cells in lymphoid organs and tissues, in a cell-intrinsic manner [18].

This prompted us to investigate the role of PD-1 in homeostasis of MP CD4 T cells. Our results indicate that PD-1 intrinsically regulates the number of MP CD4 T cells, and especially the T_EM_ subset most likely by promoting their apoptosis. PD-1 KO MP CD4 T cells were potent IFN-γ, but relatively poor IL-17A, producers. Interestingly, PD-1 also inhibits differentiation of LIP-memory CD4 T cells towards the T_EM_-phenotype.

## Material and Methods

### Mice

PD-1 KO [19], hCD2- GFP-transgenic mice (GFP) [20], hCD2-DsRed-transgenic mice (DsRed) [21], have been previously described. All mice were backcrossed to the C57BL/10 background for 10 generations. C57BL/10 (referred to as wild type, WT) and C57BL/10.PD-1-deficient mice (PD-1 KO) were used in the present study. Mice were maintained in the Institute of Molecular Biology and Biotechnology (IMBB) colony. All experiments were approved by the General Directorate of Veterinary Services, Region Crete (permit numbers: EL 91BIO-02, EL91-BIOexp-02). All efforts were undertaken to minimize animal suffering.

### Flow cytometry

Cells from spleen, thymus, lymph nodes and blood were prepared for flow cytometry as previously described [22]. The following antibodies, as well as Annexin V-FITC and propidium iodide (PI), were purchased from BD Pharmigen ^TM^: rat anti-CD4-APC (GK1.5), rat anti-CD4- PerCP (GK 1.5), rat anti-CD8b-APC (eBioH35-17.2),Armenian hamster anti-CD69-PE (H1.2F3), rat anti-CD62L-PE (MEL-14), rat anti-CD62L-PE-Cy7 (MEL-14), rat anti-CD62L-FITC (MEL-14), rat anti-CD44-PerCP-Cy5 (YM7), anti-CD44-PE, rat anti-CD25-PE (PC61), rat anti-IFN-γ-PE (XMG 1.2), rat anti-IL-2-PE (JE56-5H4), rat anti-TNF-α-PE (MP6-XT22). (J). Rat anti-IL-17A-PE (TC11-18H10.1) and rat anti-PD-1-APC (29F.1A12) was purchased by BioLegend. Rat anti-CD127-PE (A7R34), rat anti-Ki-67-PE (SOlA15), and mouse anti BrdU (5-bromo-2'-deoxyuridine)-APC (3D4) were from eBioscience. All antibodies were used according to the manufacturer’s instructions. Acquisition was carried out on a FACSCalibur or Dako Cytomation MoFlo (for five color-analysis) and data were analyzed with WinMDI or FlowJo software. Analysis was confined to live cells as judged by forward and side scatter (FSC-SSC) and occasionally evaluated by PI staining. The significance of all data was evaluated using Origin software. Student’s t-test was used in all cases except one case where Mann-Whitney test was utilized and it is explicitly stated in the corresponding figure legend. Where significant, p values are shown.

### BrdU incorporation and Ki-67 analysis

7 mo old PD-1 KO and WT mice were fed daily with 0.8 mg/ml of BrdU (Sigma) for one week. On day 7 the mice were sacrificed and splenocytes were stained as above. For BrdU analysis, cells were treated as previously described [23]. Briefly, cells were treated with FACS Lysing Solution (BD Biosciences), followed by overnight fixation in 1% paraformaldehyde containing solution. Cellular DNA was then denatured with 50 Kunitz units of DNase I (Sigma) before being stained with anti-BrdU (BD Biosciences). For Ki-67 analysis 7 mo mice were sacrificed and splenocytes were stained as above. Cells were then treated for 15 min with FACS Lysing Solution, followed by fixation at 4°C in 1% paraformaldehyde and 0.05% Nonidet-P40 for 30 min. Cells were then blocked with mouse Fcγ receptor (CD16/CD32, BD Biosciences) for 15 min, and then immediately stained with Ki-67 for 30 min at 4°C. Cells were then analyzed by flow cytometry.

### Isolation of lymphocytes from liver and lung

Mice were sacrificed and perfused via the left ventricle with 20 ml ice-cold PBS. Tissues were then teased over a filter. For lungs, Lympholyte-M (Cedarlane labs, CL5031) was used according to manufacturer’s instructions. Cell suspensions from livers were spun at 550g. The cell pellet was resuspended in RPMI and overlaid onto 33% (v/v) Percoll solution (Sigma) followed by centrifugation at 800 g for 30 min. Remaining cells after aspiration were washed twice with RPMI by centrifugation at 800 g for 5 min at 4 ^0^ C. Subsequent removal of red blood cells was performed by water lysis.

### In vivo or in vitro stimulation and intracellular cytokine staining

For cytokine production, splenocytes were incubated for 4 h in the presence of GolgiPlug (BD Biosciences) or Monensin (BioLegend) and 50 ng/ml of phorbol 12-myristate 13-acetate (PMA) and 500 ng/ml of ionomycin (both Sigma-Aldrich) or untreated. For all experiments, culture medium was RPMI 1640 (Biosera) supplemented with 10% FBS, 10 mM HEPES, 100 u/ml penicillin-streptomycin, 2 mM L-glutamine, 50 μM 2-ME. Cells were washed and stained for surface markers, as previously described. Then, cells were fixed and rendered permeable by using the Cytofix/Cytoperm Kit (BD Biosciences) according to manufacturer’s instructions, and subsequently stained for intracellular cytokines and analyzed by flow cytometry.

### Transfer of sorted CD4^+^ T cell subsets

CD4^+^ T cells were purified from spleen with MACS magnetic beads separation system (Miltenyi Biotec) according to manufacturer’s instructions. Purified (CD4^+^ GFP^+^ T cells were stained with anti-CD4-APC, anti- CD44 PerCP-Cy5 and anti-CD62L-PE for the purification of T_EM_ (CD4^+^CD44^hi^CD62L^lo^) or naïve cells (CD4^+^CD44^lo^) and sorted by Dako Cytomation MoFlo T High-Performance Cell Sorter (purity was consistently >95%). 1.5x10^5^ cells were then adoptively transferred into WT and PD-1 KO mice. Cell fate was analyzed after 32 or 64 d on the basis of CD62L and CD44 expression on donor-derived GFP^+^CD4^+^ cells. In the case of LIP experiments, 10^6^ purified naïve CD4^+^ cells were injected and recipients were sub-lethally (450 rads) irradiated WT mice.

### Generation of mixed bone marrow chimeras

Bone marrow was obtained from femurs of GFP-transgenic and PD-1 KO mice; mature T cells were first depleted by the use of mouse anti-CD90.2 (clone: 30-H12, BD Biosciences) plus complement (Cedarlane Labs), according to manufacturer’s instructions. Contamination of bone marrow cells with mature T cells was less than 0.1%. A mixture of 10^7^ WT and PD-1 KO bone marrow cells at a 1:1 ratio was injected intravenously into DsRed mice lethally irradiated with 950 rads. Chimeras were analyzed after 8 weeks.

## Results

### MP CD4 T cells accumulate in lymphoid organs and tissues of PD-1 KO mice and the majority of them are of a T_EM_-phenotype

**Fig 1:**
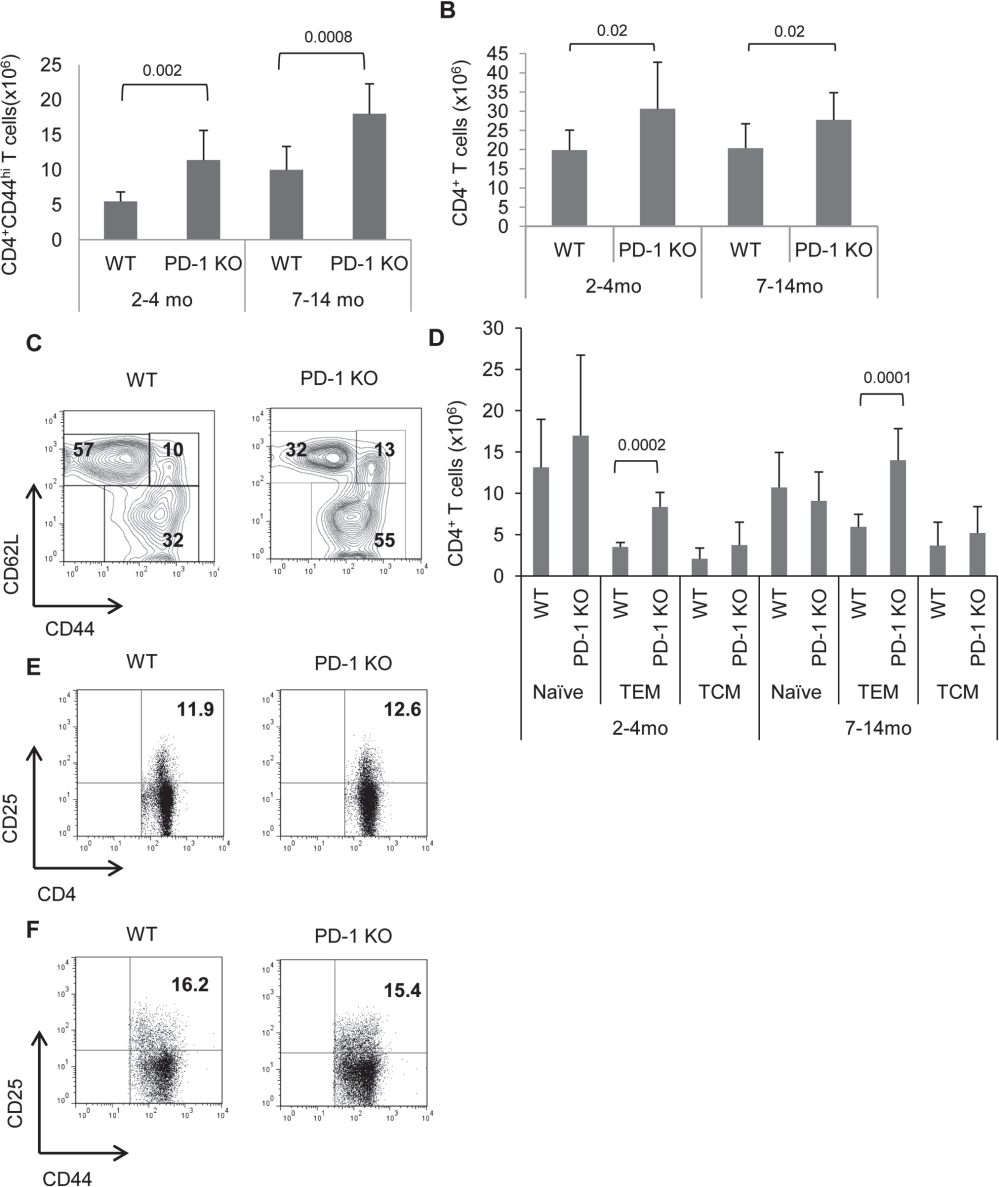
Increased numbers of CD4+ TEM-phenotype cells in spleen of PD-1 KO mice. Young (2–4 mo old) or middle-aged (7–14 mo old) mice were sacrificed and spleen cell suspensions were analyzed by flow cytometry. A, Graphs depicted average total CD4^+^CD44^hi^ spleen cell numbers of WT and PD-1 KO mice. Bars indicate mean values with error bars showing SD. B, Total CD4^+^ spleen cell numbers of WT and PD-1 KO mice at the indicated age groups. Bars indicate mean values with error bars showing SD. C,CD4^+^ splenocytes cells were further categorized phenotypically into naïve (CD44^lo^CD62L^hi^), T_CM_- (CD44^hi^CD62L^hi^), and T_EM_-phenotype (CD44^hi^ CD62L^lo^) in spleen of middle-aged mice. Representative dot plots from middle-aged mice are shown with percentages of cell subsets in each region. D, Total numbers of naïve, T_EM_ and T_CM_-phenotype CD4^+^ cells with error bars indicating the SD. Results are representative of 3 individual experiments with at least 3 mice per group. Splenocytes from PD-1 KO and WT mice were also analyzed for CD25 expression. E, Representative dot plots show expression of CD25 gated on CD4^+^ cells. F, Representative dot plots show expression of CD25 and CD44 gated on T_EM_ (CD4^+^CD44 ^hi^CD62L^hi^)-phenotype cells. Numbers indicate percentages. Results are representative of three experiments with at least two mice per group.

In order to investigate whether the MP CD4 population was affected in the absence of PD-1, we analyzed splenocytes from WT and PD-1 KO mice for the expression of CD4, and CD44 as a marker for MP T cells. As expected, the absolute number of MP CD4 T cells was found to be increased with age, both for WT and PD-1 KO spleens (Fig. 1A). Comparison between WT and PD-1 KO mice revealed significantly higher numbers of CD4^+^CD44^hi^ (MP) cells in spleens of either young or middle-aged PD-1 KO mice (Fig. 1A). It should be mentioned that in the above experiments, the total number of CD4 T cells in PD-1 KO spleens was also increased (Fig. 1B).

Next, we wanted to examine the contribution of naïve, T_EM_ and T_CM_ subsets to the CD4 T cell pool. By including CD62L in our analysis, we found a much higher proportion of CD44^hi^CD62L^lo^ cells (T_EM_) within the CD4^+^ cell compartment of PD-1 KO mice with a parallel decrease of naïve CD4 T cells proportion (Fig. 1C). When we analyzed total cell numbers for each subset we found that the only statistically significant difference was the pronounced accumulation of CD4 T_EM_ cells in PD-1 KO compared to WT (∼2.5-fold) spleens, in young and middle-aged mice (Fig. 1D).

The CD44^hi^ CD4^+^ compartment contains regulatory T cells as well. The proportion of CD25^+^ cells (which is a marker of Tregs) on CD4^+^ PD-1 KO cells was similar with WT cells (Fig. 1E). Moreover CD4^+^ T_EM_ cells from WT or PD-1 KO mice contained similar percentages of CD25^+^ cells (Fig. 1F). This indicates that increased numbers of CD4^+^ T_EM_ cells found in PD-1 KO mice are not attributed to a possible abnormal Treg population.

**Fig 2:**
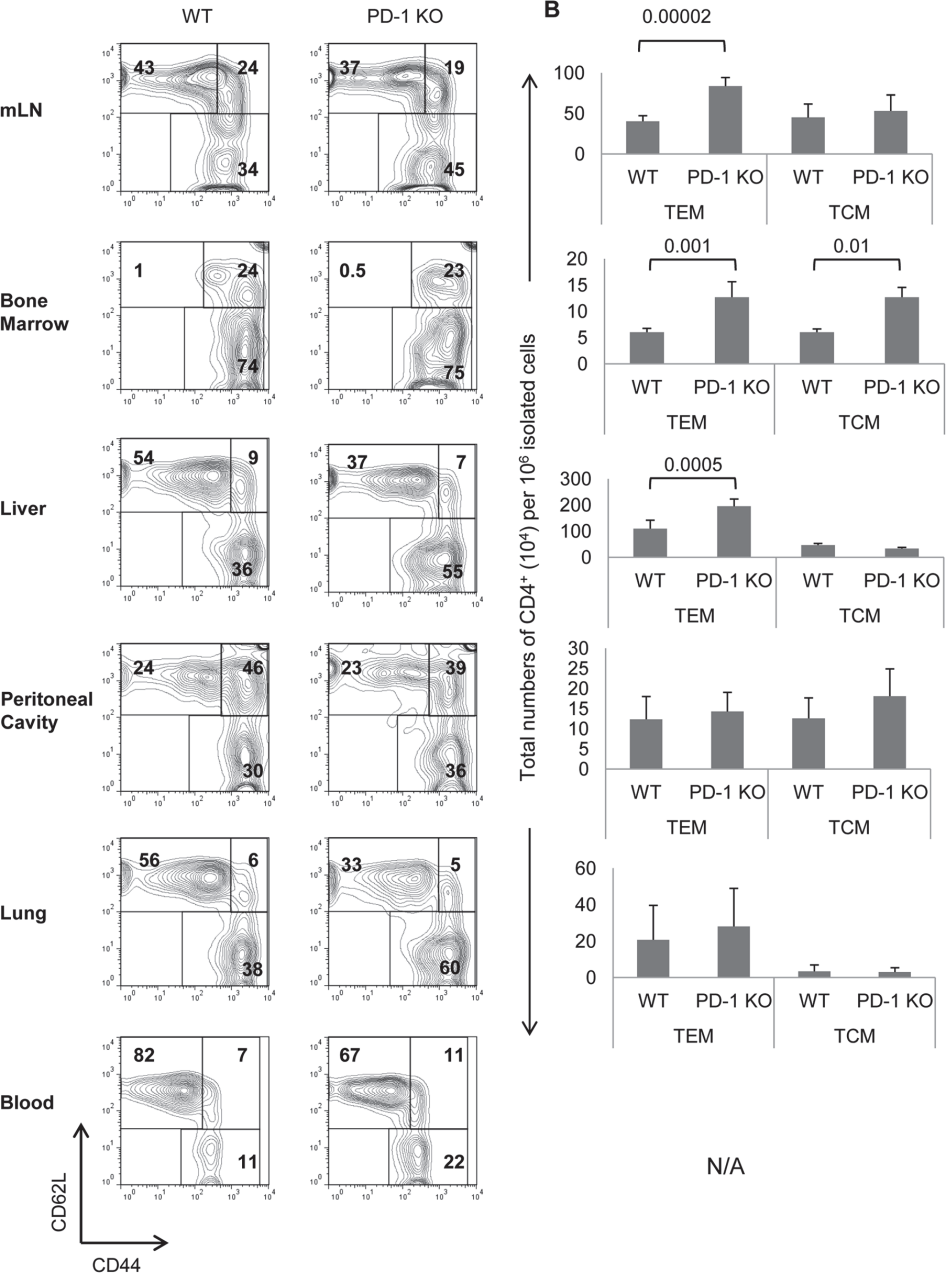
Increased numbers of TEM-phenotype CD4+ cells in lymphoid and non-lymphoid tissues of PD-1 KO mice. 9 mo old WT and PD-1 KO mice were sacrificed and cell suspensions from various lymphoid and non-lymphoid tissues were categorized phenotypically by flow cytometry into naïve (CD44^lo^CD62L^hi^), T_CM_ (CD44^hi^CD62L^hi^), and T_EM_-phenotype (CD44^hi^CD62L^lo^) CD4^+^ cells. A, Representative dot plots are shown with percentages of cells per region. B, Total numbers of CD4 T_CM_ and T_EM_-phenotype cells per 10^6^ isolated cells are shown, with error bars indicating SD. The results are representative of 3 individual experiments with at least 2 mice per group.

It could be possible that the observed accumulation was due to some alteration in the migratory properties of CD4 T_EM_ cells since PD-1 has been shown to affect T cell adhesion and potential migration [24]. Moreover, T_EM_ cells preferentially migrate to tissues and spleen and not in lymph nodes. Examination of PD-1 KO mesenteric lymph nodes for CD4 T_EM_ cells revealed pronounced accumulation of these cells compared to WT mice (Fig. 2A and B), similarly to that observed in the spleen. The same degree of increased accumulation of PD-1 KO CD4 T_EM_ cells was observed in liver and bone marrow and also in blood. Notably, in a bone marrow a significant increase of T_CM_ cells was also observed. Although higher accumulation of PD-1KO CD4 T_EM_ cells was not consistently observed in lung and peritoneal cavity, there was no single tissue or lymphoid organ that PD-1 KO T_EM_ cells were less than the WT (Fig. 2A and B).

The above results show that there is accumulation of MP CD4 T cells in lymphoid organs and some tissues of PD-1 KO mice. This is mainly due to expansion of the T_EM_ subset which cannot be attributed to dysregulated T cell migration.

### CD4 T_EM_-phenotype cells from WT and PD-1 KO mice differ in effector cytokine expression and surface markers

**Fig 3:**
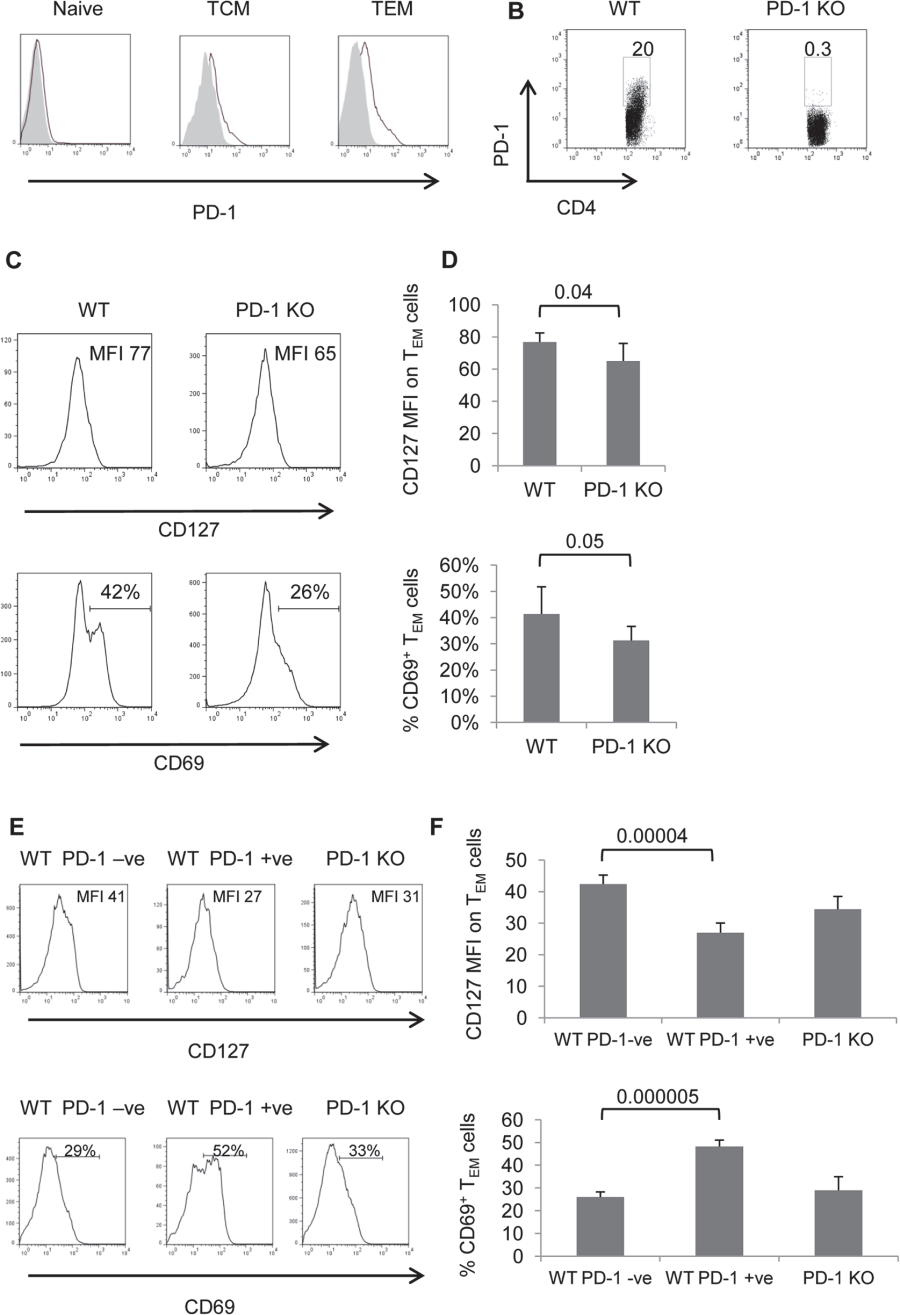
Phenotypic analysis of MP and TEM CD4 cells from WT and PD-1 KO mice. Splenocytes from 7–9 mo old PD-1 KO and WT mice were analyzed by flow cytometry. A, Representative histograms show expression of PD-1 gated on CD4 T cells subsets (shaded histogram: PD-1 KO, thick line: WT). Data are representative of 2 individual experiments (n = 7). B, Representative dot blots depict PD-1 expression on CD4 T_EM_ cells, number indicate percentages in region. C, Representative dot plots show expression of CD127 and CD69 gated on T_EM_ (CD4^+^CD44^hi^CD62L^lo^)-phenotype cells. D, Graphs depict averages of mean fluorescence intensity (MFI) for CD127 expression (upper panel) and percentage of CD69^+^ cells (lower panel) on T_EM_-phenotype (CD44^hi^CD62L^lo^) CD4^+^ cells. Data represent 2 individual experiments with 3 or 4 mice per group. E, Representative histograms illustrate expression of CD127 (upper panels) and CD69 (lower panels) gated on WT PD-1 negative (-ve), PD-1 positive (+ve) and PD-1 KO T_EM_-phenotype cells. F, Graphs depict averages of mean fluorescence intensity (MFI) for CD127 expression (upper panel) and percentage of CD69^+^ cells (lower panel) on WT PD-1 negative (-ve), PD-1 positive (+ve) and PD-1 KO T_EM_-phenotype CD4^+^ T cells. Error bars indicate SD. Data represent 2 individual experiments with 3 or 4 mice per group.

We analyzed PD-1 expression on naïve, T_CM_ and T_EM_ CD4 cells. No PD-1 expression was observed on WT naïve cells, while its expression was more prominent on CD4 T_EM_ cells (Fig. 3A). This is in agreement with previous observations in C57BL/6 mice [25]. Approximately 20% of CD4 T_EM_ cells express PD-1 on their surface (Fig. 3B).

In line with the observation regarding T_EM_ cell accumulation we phenotypically characterized CD4 T_EM_ cells from WT and PD-1 KO mice. IL-7 is an important survival factor for memory CD4 T cells [26] and expression of its receptor allows cells to utilize the cytokine. Analysis on WT and PD-1 KO CD4 T_EM_ cells revealed that PD-1 KO CD4 T_EM_ cells express slightly, but significantly, lower levels of surface IL-7Rα (CD127) (Fig. 3C and D). Five color analysis revealed that the PD-1-ve fraction of WT CD4 T_EM_ cells expressed higher levels of IL-7Rα compared to PD-1 +ve cells (Fig. 3E and F). CD69 is an early activation marker for T cells and its expression is associated with the generation and maintenance of antigen-specific memory CD4 T cells [27], and also CD69 inhibits CD4 cell egression from lymphoid organs to the tissues [28]. A smaller percentage of PD-1 KO CD4 T_EM_-phenotype cells are CD69^+^ compared to WT counterparts (Fig. 3C and D). However the relevance of this is not clear, since CD4-T_EM_ phenotype cell numbers in PD-1 KO lymphoid organs are higher than WT. Five color analysis showed that a larger proportion of PD-1 +ve WT CD4 T_EM_ cells expressed CD69 when compared to PD-1-ve counterparts (Fig. 3E and F).

**Fig 4:**
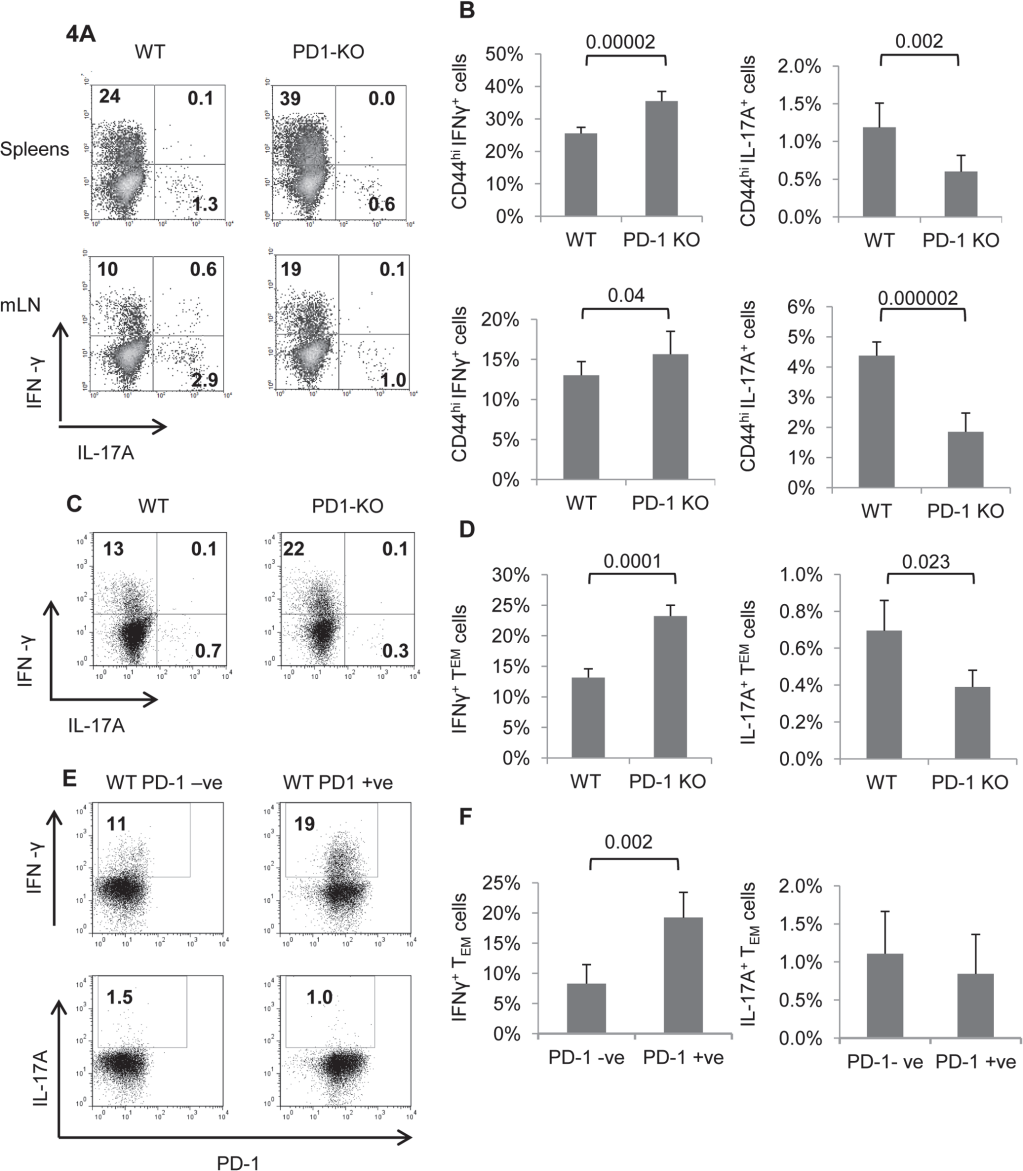
Functional analysis of MP and TEM CD4 cells from WT and PD-1 KO mice. A, Cells from spleens and mesenteric lymph nodes of 7 mo old PD-1 KO and WT mice were analyzed by flow cytometry, after brief *ex vivo* stimulation with PMA and ionomycin. Representative dot plots of IFN-γ and IL-17A producing cells gated on CD4^+^ CD44^hi^ cells. B, Graphs depict average percentages of IFN-γ and IL-17A producing CD4^+^CD44^hi^ cells from spleen and mesenteric lymph nodes. Error bars indicate SD. Data represent 2 individual experiments with 4 mice per group. C, Representative dot plots of IFN-γ and IL-17A production by purified CD4 T_EM_ (CD4^+^CD44^hi^CD62L^lo^)-phenotype cells. D, Graphs illustrate average percentages of IFN-γ and IL-17A production as in C. Error bars indicate SD. Data represent two individual experiments with eight pooled spleens per group. E, Representative dot plots of IFN-γ (upper panel) and IL-17A (lower panel) production by purified PD-1 negative (-ve) and PD-1 positive (+ve) CD4 T_EM_-phenotype cells. F, Graphs illustrate average percentages of IFN-γ and IL-17A production as in E.

Decreased IL-7Rα and increased CD69 expression is consistent with previous findings in PD-1^+^ CD4 cells from C57BL/6 mice [29]. Memory phenotype CD4 T cells have typical properties of memory cells including rapid production of IFN-γ [4] or IL-17A [30]. Production of these cytokines is considered as a hallmark of their function and is related to the commitment of CD4 T cells upon differentiation to Th1 or Th17 lineages respectively [31]. To assess whether the lack of PD-1 during the differentiation and/or reactivation period perturbed the ability of MP CD4 T cells to produce effector cytokines, we briefly challenged splenocytes with PMA and ionomycin *ex vivo*, and assayed for intracellular IFN-γ and IL-17A production by MP CD4 cells. For both WT and PD-1 KO mice IL-17A^+^ cells were more abundant among CD4^+^CD44^hi^ (MP) cells from mesenteric LNs compared to spleen, whereas IFN-γ^+^ cells were more abundant in the spleen (Fig. 4A and B). Importantly, these experiments showed that a significantly higher proportion of MP CD4 PD-1 KO cells was able to produce IFN-γ compared to WT in spleen and to a lesser extent in mesenteric LNs (Fig. 4A and B). Conversely, a much lower proportion of IL-17A^+^ cells was identified among PD-1 KO MP CD4 T cells (Fig. 4A and B). No significant difference was found in the proportion of WT and PD-1 KO MP CD4 T cells that were able to produce IL-2 and TNF-α (S1 Fig.).

Reciprocal regulation of IFN-γ and IL-17A production by PD-1 was also evident when we challenged purified (CD4^+^CD44^hi^CD62^lo^) WT and PD-1 KO CD4 T_EM_ cells *ex vivo*. Specifically, a higher proportion of PD-1 KO CD4 T_EM_ cells was able to produce IFN-γ while a smaller percentage of these cells was able to produce IL-17A when compared to WT counterparts (Fig. 4C and D).

Interestingly, in a separate set of experiments, we found that among WT T_EM_ cells, the purified PD-1 +ve fraction bore more IFN-γ^+^ cells relative to the PD-1-ve fraction, indicating that in these particular settings PD-1 expression was not correlated with an “exhausted” phenotype. No significant differences in IL-17A production was observed between the two fractions (Fig. 4E and F).

Overall, these results indicate that PD-1 regulates the differentiation of CD4 T cells to memory phenotype cells, and particularly T_EM_ cells, capable of producing IFN-γ and/or IL-17A.

### Reduced apoptosis but not increased homeostatic proliferation can account for the accumulation of PD-1 KO CD4 T_EM_-phenotype cells

MP CD4 T cells differentiate from naïve cells through homeostatic proliferation in response to self-ligands [1], or through reactivity against environmental antigens [4] although in mice the latter mechanism is disputable [32]. It is possible that PD-1 mutation affected the T_EM_ subset per se and is not involved in above mentioned differentiation towards T_EM_ cells. In that case, PD-1 KO T_EM_ cells would exhibit increased survival potential and/or increased proliferation rates.

**Fig 5:**
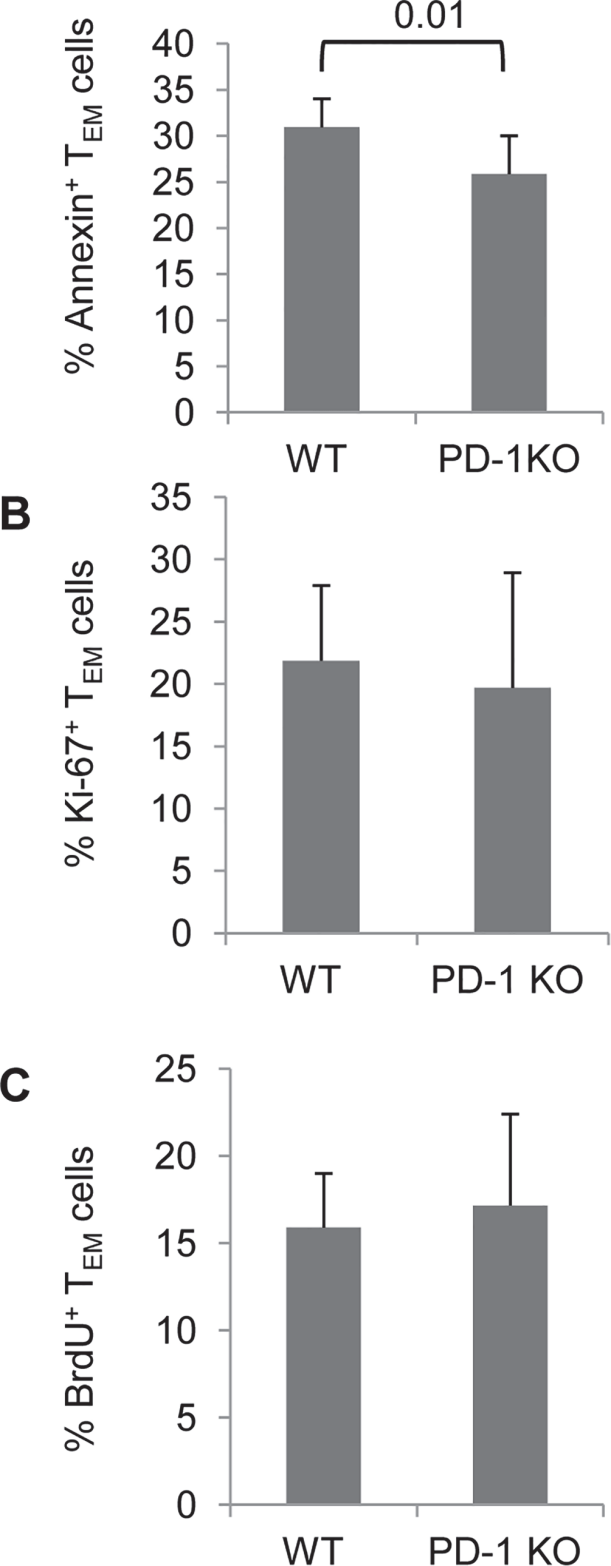
Analysis of apoptosis and homeostatic proliferation on TEM-phenotype cells. Splenocytes from 7 mo old PD-1 KO and WT mice were analyzed for Annexin V binding, Ki-67 expression and BrdU incorporation. A, Mean percentages of Annexin V^+^ cells among T_EM_-phenotype CD4 cells, gated on live cells as confirmed by propidium iodide staining. B, Mean percentages of Ki-67^+^ and C, BrdU^+^ cells among T_EM_-phenotype CD4 cells. Data represent 2 individual experiments with 4 mice per group. Error bars indicate SD.

Upon analyzing Annexin V-binding on freshly isolated splenocytes we found a smaller percentage of Annexin^+^ cells among PD-1 KO CD4 T_EM_-phenotype cells in comparison to WT counterparts (Fig. 5A). For analysis of in vivo proliferation, we assessed Ki-67 expression and we found no differences in CD4 T_EM_-phenotype cells between WT and PD1 KO group (Fig. 5B). We also analyzed BrdU incorporation in T_EM_ cells after feeding to mice and no consistent difference was found (Fig. 5C).

These results indicate that decreased apoptosis, but not increased proliferation, can account -at least partly- for the accumulation of CD4 T_EM_-phenotype cells in PD-1 KO mice.

### PD-1 KO CD4 T_EM_-phenotype cells differentiate from peripheral naïve CD4 T cells

**Fig 6:**
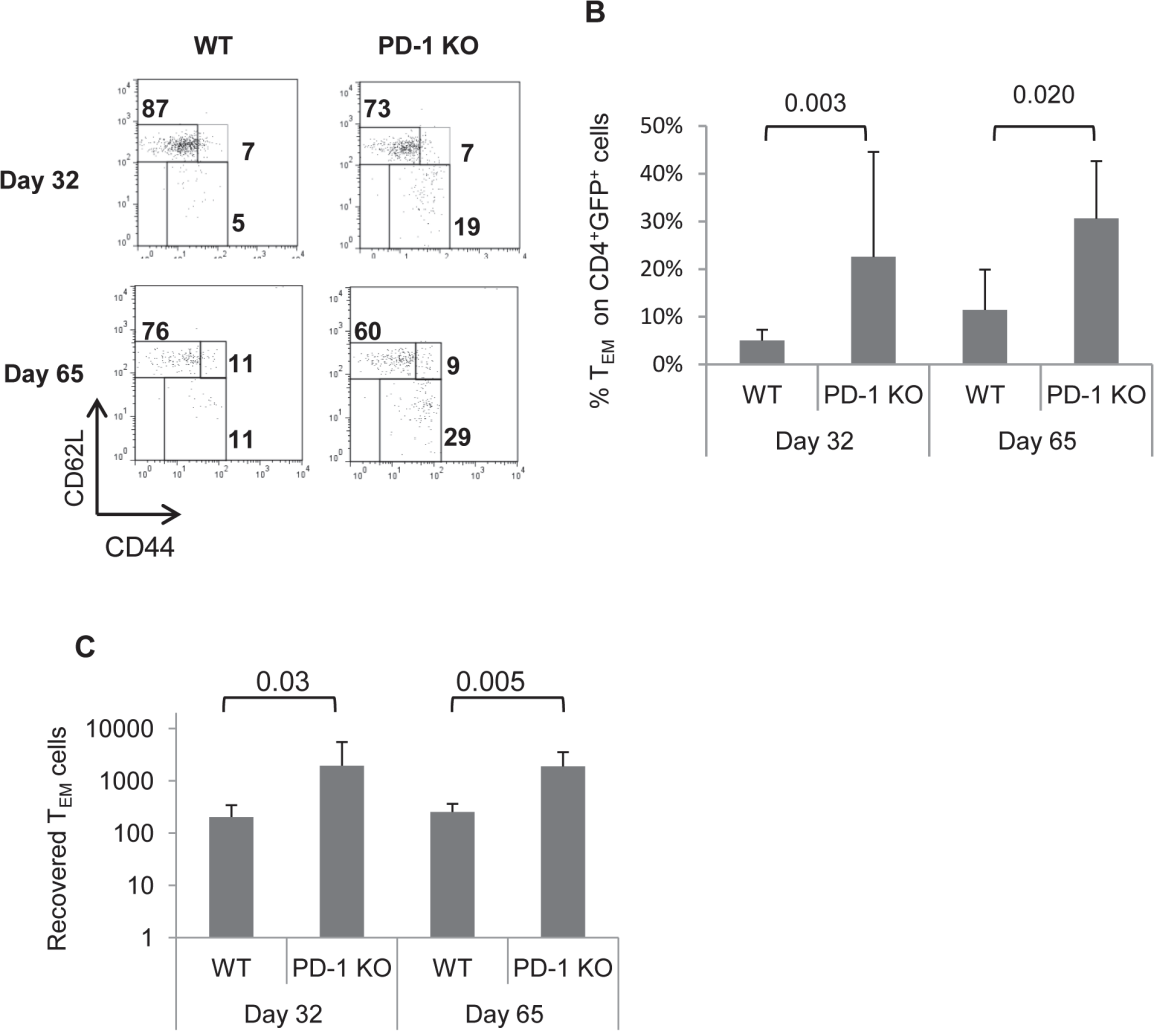
Fate of naïve WT and PD-1 KO CD4+ cells transferred into WT and PD-1 KO mice. GFP^+^CD4^+^CD44^lo^ cells from spleens of 4 mo old WT or PD-1 KO mice were purified by FACS sorting. Cells were then adoptively transferred into WT or PD-1 KO mice respectively. On Day 32 or 65, mice were sacrificed and spleens were analyzed.A, GFP+donor-derived cells were analyzed for CD4, CD44 and CD62L expression and representative dot plots are shown. B. Graphs display the mean percentage of T_EM_-phenotype cells as in A. C, Graphs depict total numbers of GFP^+^ T_EM_ CD4^+^ T cell found in host spleens at each time point. Data are representative of 3 individual experiments (n = 9). Error bars indicate SD. Statistical significance was evaluated with Mann-Whitney test.

Normally, CD44^hi^CD62L^lo^ CD4 T cells are considered effector-memory phenotype cells of peripheral origin i.e. their progenitors had been peripheral naïve CD4 T cells. However, under certain circumstances, CD44^hi^CD62L^lo^ CD4 T cells may have a thymic origin [33]. We aimed to investigate the possibility whether PD-1 mutation promotes accumulation of thymus-derived CD44^hi^CD62L^lo^ CD4 T cells, by transferring purified GFP.WT or GFP.PD-1 KO CD4^+^ peripheral naïve cells to normal WT or PD-1 KO hosts, respectively. Moreover, in these settings we would be able to monitor the rise of CD4 T_EM_ cells with progression of time. After 32 days, a small percentage of GFP.WT CD4 donor-derived cells belonged to the T_EM_ subset (with an average of 5,05%) while the respective frequency of GFP.PD-1 KO T_EM_ cells was higher (with an average of 22,6%) (Fig. 6A, upper panel, and 6B, first and second columns) but with substantial variability among mice. As time progressed (65 days) the frequency of GFP.WT CD4 T_EM_ cells rose to an average of 14.7% where GFP.PD-1 KO counterparts rose to 30,6% (Fig. 6A, lower panel, and 6B, third and fourth columns). Calculation of total recovered T_EM_-cells at both time points revealed a significantly higher number of GFP^+^ CD4 T_EM_ cells when donors and acceptors were PD-1 KO (Fig. 6C). No significant difference was observed between WT and PD-1 KO mice regarding numbers of naïve or T_CM_ GFP.CD4 cells (not shown). However, from these experiments we do not have evidence whether T_EM_ cells increase at the expense of other subsets, due to variability in cell recovery among mice. Notably, reduced cell death of PD-1 KO T_EM_ cells that we described in Fig. 5A remains a valid explanation.

Nonetheless, these results strongly indicate that CD44^hi^CD62L^lo^ (T_EM_-phenotype) cells accumulating in PD-1 KO mice differentiate from peripheral naïve CD4 T cells and they are not solely of thymic origin.

### Absence of PD-1 favors differentiation of CD4 T_EM_ cells through lymphopenia-induced proliferation

**Fig 7:**
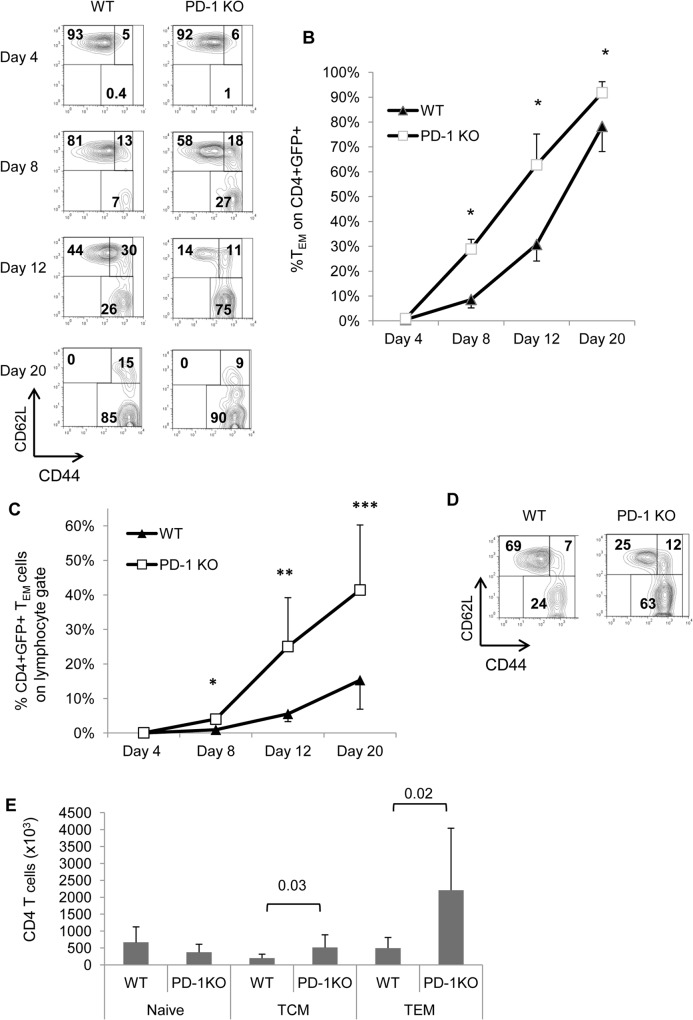
LIP of PD-1 KO naive CD4 T cells promote TEM-phenotype cell differentiation. Naive CD4^+^GFP^+^ CD44^lo^ cells from spleens of 2–4 mo old PD-1 KO and WT mice were isolated by FACS sorting. Purified cells were then adoptively transferred into sublethally irradiated WT mice. A, GFP^+^ CD4^+^ cells in hosts’ blood were examined for the expression of CD4, CD44 and CD62L on days 4, 8 and 12 and 20. Numbers indicate percentages in each region. Data are representative of 2 experiments with 3 mice per group. B, Graph diplays the average percentage of T_EM_-phenotype cells within donor-derived CD4^+^GFP^+^ cells in blood at various time points as in A. Error bars indicate SD. C, Frequency of donor-derived T_EM_ (CD4^+^CD44^hi^CD62L^lo^)-phenotype cells within lymphocyte gates at various time points in blood of recipient mice (* = 0.001, ** = 0.02, *** = 0.0007). D, On Day 20, mice were sacrificed and spleens were analyzed as in A. Numbers indicate percentages in each region. E, Total numbers of donor-derived GFP^+^ CD4 T cell subsets found in spleens of host mice. Error bars indicate SD. Plots are representative of 3 individual experiments. (WT, n = 11; PD-1 KO, n = 10).

CD4 T lymphocytes with a phenotype similar to MP cells can arise through lymphopenia-induced-proliferation (LIP-memory cells) [34] and co-stimulatory molecules have been shown to interfere with this process [35]. To examine the possibility that PD-1 deficiency regulates differentiation of LIP-memory CD4 T cells, we transferred purified peripheral naïve GFP.WT or GFP.PD-1 KO CD4 cells to sublethally irradiated WT recipients. We monitored the emergence of LIP-memory cells with time in recipients by analyzing blood samples at certain time points. At the earliest time point (Day 4), only few CD44^+^ LIP-memory cells have been formed and, among them, practically no donor-derived T_EM_- phenotype cells were found (Fig. 7A). On Day 8 about 9% WT donor-derived GFP^+^CD4^+^ cells had a T_EM_-phenotype whereas almost 30% of PD-1 KO derived GFP^+^CD4^+^ cells had differentiated to T_EM_ cells (Fig. 7A and B). On Day 12, CD4 T_EM_-phenotype cells from PD-1 KO donors had already outnumbered naïve cells whereas the dominant fraction of WT-derived cells still had a naïve phenotype (Fig. 7A). On Day 20, both WT- and PD-1 KO-derived cells were mostly T_EM_-phenotype, but the proportion of T_EM_-phenotype cells was consistently higher when donor cells were of PD-1 KO origin (Fig. 7A and B). We also calculated the percentage of CD4 T_EM_-phenotype cells within blood lymphocyte gate (which reflects their total number) and we found that on Days 8, 12, and 20 the frequency of PD-1 KO CD4 T_EM_ cells within lymphocyte gate was 3–5 times higher as compared to the frequency of their WT counterparts (Fig. 7C).

Similar findings at Day 20 were observed in spleen, where analysis revealed that the total numbers of CD4 T_EM_ LIP-memory cells were significantly higher in the spleens of mice which had received PD-1 KO naïve cells (Fig. 7D and E).

These results show that PD-1 negatively regulates the formation of T_EM_-phenotype from naïve CD4 cells through lymphopenia-induced proliferation.

### Cell-intrinsic mechanisms account for the accumulation of PD-1 KO CD4 T_EM_-phenotype cells

**Fig 8:**
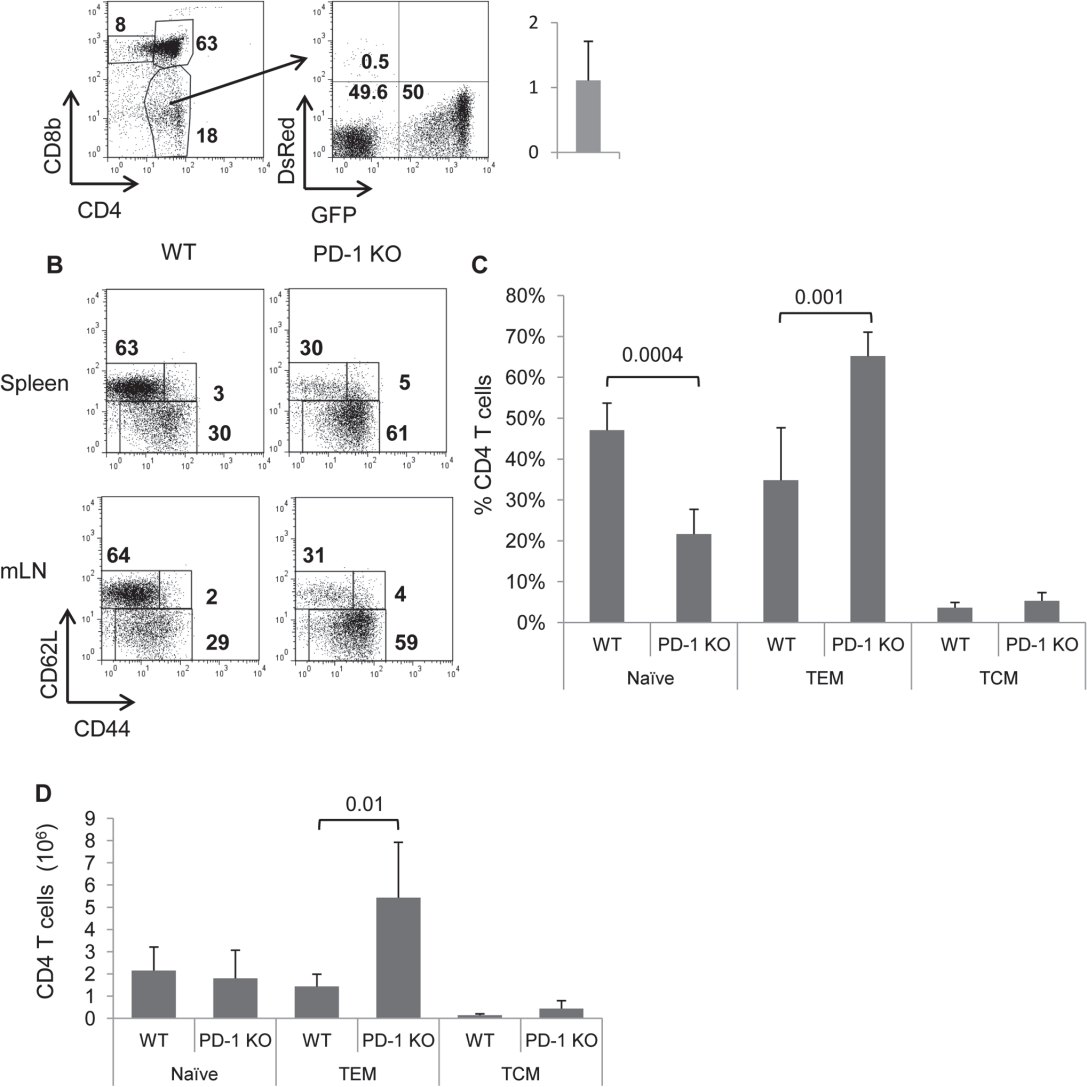
T cell-intrinsic increase in PD-1 KO CD4+ TEM-phenotype cells. Cells from thymi, spleens and lymph nodes were analyzed by flow cytometry for donor-derived WT (GFP^-^DsRed^-^) and PD-1 KO (GFP^+^DsRed^-^) CD4^+^ cells 8 weeks after bone marrow reconstitution in lethally irradiated DsRed hosts. A, Dot plots show donor-derived WT (GFP^-^DsRed^-^) and PD-1 KO (GFP^+^DsRed-) thymocytes. On thymocytes the expression of CD4 and CD8 was analyzed (left). The distribution WT GFP^-^ and GFP^+^ PD-1 KO cells were assessed among CD4 SP cells (right). Numbers indicate percentages in each region. Column represents the average value of PD-1 KO/WT CD4 SP thymocyte ratios with error bar indicating SD. In one of the thymus experiments the WT donor cells were GFP^+^ and PD-1 KO cells were GFP^-^. B, Donor-derived WT (GFP^-^DsRed^-^) and PD-1 KO (GFP^+^DsRed^-^) CD4^+^ T cells from spleens and mesenteric lymph nodes were further analyzed for expression of CD44 and CD62L. Numbers indicate percentages in each region. C, Graph indicates the average percentage of cell subsets among WT- and PD-1 KO-derived CD4^+^ cells in spleens, as in B. Error bars denote SD. D, Total numbers of WT- and PD-1 KO-derived CD4^+^ cell subsets in spleens with error bars indicating SD. Data are representative of 2 individual experiments, n = 6.

To determine whether accumulation of CD4 T_EM_ cells in PD-1 KO mice is cell intrinsic, we performed mixed bone marrow chimera transplantation experiments. In particular, we transferred mixtures consisting of equal numbers of WT and GFP.PD-1 KO bone marrow cells to lethally irradiated DsRed.WT recipients. After 8 weeks, thymi, spleens, and lymph nodes were analyzed for GFP, DsRed, CD4, CD44, and CD62L expression. Flow cytometric analysis revealed a similar representation of WT (GFP^-^) and PD-1 KO (GFP^+^) cells among CD4 SP thymocytes (Fig. 8A). This indicated that the PD-1 mutation does not confer any obvious advantage or disadvantage to developing CD4 T cells. Interestingly, in both spleens and mesenteric lymph nodes, as shown in Fig. 8B and C a substantially higher percentage of T_EM_-phenotype cells was found among CD4 T cells of GFP. PD-1 KO origin compared to WT (GFP^-^), similar to what we observed in normal PD-1 KO mice (Fig. 1C). This was also reflected in the significantly higher numbers of PD-1 T_EM_-phenotype cells recovered from spleen of recipient mice (Fig. 8D). The fact that both WT and PD-1 KO cells developed in the same environmental cues strongly indicates that the reason for accumulation of PD-1 KO T_EM_ cells is cell intrinsic.

## Discussion

In this study we demonstrated that the absence of PD-1 expression leads to accumulation of MP CD4 T cells and specifically T_EM_-phenotype cells. This was prominent in spleen, lymph node and some of the organs examined. In none of the tissues of PD-1 KO mice examined did we find less CD4 T_EM_ cells, as compared to WT mice, which supports the hypothesis that the effect is not related to a consistent, global alteration of migration patterns of MP CD4 cells due to lack of PD-1. An obvious explanation would be a higher rate of homeostatic proliferation in the absence of PD-1 which we formally excluded by assaying Ki-67 expression or BrdU incorporation (Fig. 5B and C). However, we observed lower rates of apoptosis in PD-1 KO CD4 T_EM_-phenotype cells in vivo (Fig. 5A). Memory cells rely on IL-7 for survival [36] and analysis of IL-7Rα expression did not reveal a possible explanation for the decreased apoptosis of PD-1 KO T_EM_-phenotype cells (Fig. 3C and D). Similarly, Bcl-2 expression was not consistently different between WT and PD-1 KO T_EM_ cells (not shown). It is possible that other intrinsic factors contribute to the survival advantage of PD-1 KO T_EM_ cells.

CD69^+^ cells were more frequent among PD-1 KO CD4 T_EM_-phenotype cells compared to WT (Fig. 3C and D) and one could argue that these cells represent recently activated effector cells rather than effector memory. However, the similar expression of CD25 between WT and PD-1 KO T_EM_ cells (Fig. 1F) as well as the comparably low proliferative rate, determined by Ki-67 expression and BrdU-incorporation experiments (Fig. 5B and C), disfavor this scenario.

Cells with similar phenotypic characteristics (CD4^+^CD44^hi^CD62L^lo^) have been described to originate from the thymus [33]. Although we did not exclude that some of the accumulated T_EM_-phenotype have a thymic origin, our evidence strongly suggests that the expanded population of T_EM_-phenotype cells in PD-1 KO mice originates from peripheral naïve cells. First, we transferred purified peripheral naïve CD4^+^ cells to lymphosufficient hosts and after 32 or 65 days, a significant proportion of them had acquired the T_EM_-phenotype (Fig. 6). Second, transfer of purified naïve CD4 T cells to lymphodeficient hosts resulted in very high percentages of T_EM_-phenotype cells, especially when donors were lacking PD-1 (Fig. 7).

Memory-like CD4 T cells can be generated in response to T cell lymphopenia (LIP-memory). LIP-memory cells exhibit functional properties similar to antigen-primed T cells [5]. We showed that PD-1 mutation also may affect differentiation and/or survival of LIP-memory cells. Specifically, transfer of naïve CD4 cells and the subsequent LIP led to a progressive emergence of T_EM_-phenotype cells in blood which was faster and more prominent in mice that received PD-1 KO cells (Fig. 7A-C). Accordingly, PD-1 KO T_EM_-phenotype cells recovered from host spleens at the end point were 4–5 times more abundant than WT ones (Fig. 7D and E). It is of notice that T_CM_ cells differentiate first (already present on Day 4) followed by the emergence of T_EM_ cells (from Day 8 and onwards). This, in combination with the observation that a CD44^hi^CD62L^int^ population is evident (Days 8, 12, 20 especially for PD-1 KO donors), suggests that T_EM_-phenotype cells differentiate through a T_CM_ intermediate. This was shown to be the case for CD8 T_EM_-phenotype cells where the PD-1 mutation promoted the conversion of T_CM_- to T_EM_-phenotype cells [18]. The fact that PD-1 pathway impedes accumulation of CD4 and CD8 T_EM_ cells during LIP may be important in regulating autoimmunity correlated with lymphopenia settings [37].

Memory CD4 T cell homeostasis is affected by extrinsic factors such as IL-7 or IL-2 [38,39,40]. It would be possible that PD-1 mutation had an effect on other cell types to produce factors that are involved in the differentiation or survival of CD4 T_EM_-phenotype cells. Our data with mixed bone marrow chimera transfers showed that when WT and PD-1 KO CD4 cells undergo all the differentiation steps in the same environment, there is a post-thymic selective propensity for accumulation of PD-1 KO T_EM_-phenotype cells (Fig. 8) which disfavors the contribution of extrinsic factors. The intrinsic nature of this accumulation is further supported by the generation of larger numbers of LIP-memory CD4 T_EM_ cells, in the absence of PD-1 only by donor CD4 T cells (Fig. 7).

Co-stimulation seems to be an important determinant in differentiation towards T_EM_-phenotype cells. In particular for CD4 T cells, the positive co-stimulatory molecules ICOS [11,41], and OX-40 [12] promote accumulation of CD4 T_EM_-phenotype cells. This is supportive of the hypothesis that increased signal strength augments the differentiation to T_EM_ cells [42]. In line with the above hypothesis, we provide evidence that the co-receptor PD-1 puts a break on the differentiating MP CD4 cells. Upon the removal of this break, signal strength becomes stronger and skews the developing MP cells towards a T_EM_ phenotype.

Effector and memory CD4 T cells differentiate upon activation to certain Th lineages which are characterized by production of “fingerprint” cytokines. *Ex vivo* analysis of cytokine production revealed that, compared to WT, a larger fraction of PD-1 KO CD4 MP cells belong to the Th1 phenotype and a smaller to the Th17 phenotype as judged by their ability to produce IFN-γ and IL-17A respectively (Fig. 4A and B). Similar differential regulation of IFN-γ and IL-17A production by PD-1 was observed for isolated CD4 T_EM_ cells (Fig. 4C and D). This stimulatory effect of PD-1 ablation on Th1 cells has been shown in the late phase of infection of mice with *M*.*bovi*. [43]. Accordingly, ablation of PD-1 pathway contributes to IFN-γ production and functional restoration of CD4 T cells, correlated with clearance of blood stage malaria in mice [44]. Additionally, PD-1 blockade on lymphocytic choriomeningitis (LCMV)-specific CD4 T cells rejuvenated exhausted virus specific CD8 T cells and reduced viral load [45]. Importantly, high levels of IFN-γ production by memory CD4 T cells may also be critical for protection against influenza in humans [46], which implies a possible role for manipulating PD-1 pathway in vaccine regimens. It is intriguing, however, that there were more IFN-γ producers among the PD-1 +ve fraction of WT CD4 T_EM_ cells relative to the PD-1-ve (Fig. 4E and F). A possible explanation could be that the PD-1 KO CD4 T_EM_ population is not identical to the PD-1-ve WT CD4 T_EM_ population. Production of IL-2 and TNF-α was similar on a per cell basis between WT and PD-1 KO MP CD4 T cells (S1 Fig.); however if we consider the increased numbers of these cells in PD-1 KO mice, the potential for IL-2 and TNF-α production during an immune response was higher in knock-out animals.

Collectively, our results show that PD-1 pathway determines the composition and function potential of memory-phenotype CD4 T cell pool. This may be taken in consideration in vaccine design as well as in treatment of chronic infections. The T_CM_/T_EM_ ratio of memory CD4 T cells and their function can also be critical in determining T cell responses to cancer cells [47]. There is also mounting evidence for the involvement of memory CD4 T cells, and especially T_EM_ cells, in the pathogenesis of autoimmune diseases such as systemic lupus erythematosus and in autoimmune diabetes [10]. Therefore, a better understanding of the factors that govern the accumulation of these cells, could lead to possible new therapeutic interventions.

## Supporting Information

S1 Fig IL-2 and TNF-α production by CD4 T_EM_-phenotype cells. Spleens from 7–9 mo old PD-1 KO and WT mice were analyzed by flow cytometry, after brief ex vivo stimulation with PMA and ionomycin. Representative histograms of IL-2 and TNF-α production, gated on CD4^+^CD44 ^hi^CD62L^hi^ T_EM_-phenotype. Numbers indicate percentages. Data are representative of 2 individual experiments with 4 mice per group.
(TIF)
