# Supplementary figures and images for: Programmed Death 1 Regulates Memory Phenotype CD4 T Cell Accumulation, Inhibits Expansion of the Effector Memory Phenotype Subset and Modulates Production of Effector Cytokines
Source: PLoS One. 2015 Mar 24;10(3):e0119200. doi: 10.1371/journal.pone.0119200 (PMC4372408; doi:10.1371/journal.pone.0119200)

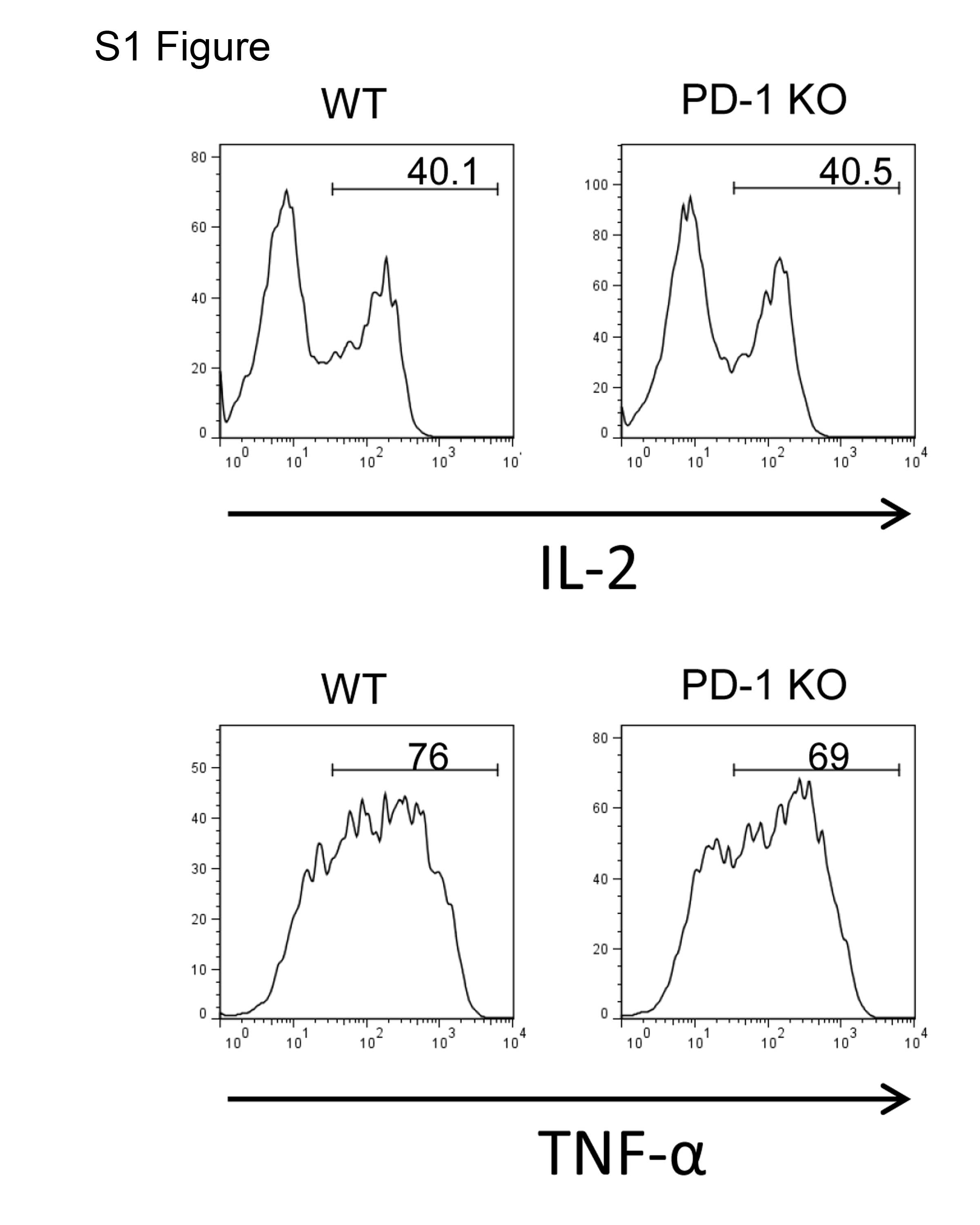

Supplement: S1 Fig — Spleens from 7–9 mo old PD-1 KO and WT mice were analyzed by flow cytometry, after brief ex vivo stimulation with PMA and ionomycin. Representative histograms of IL-2 and TNF-α production, gated on CD4+CD44 hiCD62Lhi TEM-phenotype. Numbers indicate percentages. Data are representative of 2 individual experiments with 4 mice per group. (TIF) [file pone.0119200.s001.tif]
